# The intestinal fatty acid binding protein-2 Ala54Thr polymorphism is associated with diabetic retinopathy in Chinese population

**DOI:** 10.1186/s13098-015-0021-8

**Published:** 2015-03-21

**Authors:** Zhu Li, Chang-Lin Ni, Wen-yan Niu, Bao-cheng Chang, Li-Ming Chen

**Affiliations:** Key Laboratory of Hormone and Development, Ministry of Health, Tianjin Metabolic Disease Hospital, Tianjin Medical University, Tianjin, 300070 China; Department of Immunology, Key Laboratory of Immuno Microenvironment and Disease of the Educational Ministry of China, Tianjin Medical University, Tianjin, 300070 China

**Keywords:** The intestinal fatty acid binding protein-2, Diabetic retinopathy, Single nucleotide polymorphism

## Abstract

**Objective:**

Endothelial dysfunction which is induced by serum saturated fatty acids increasing is one of pathogenesis of diabetic retinopathy (DR). The intestinal fatty acid binding protein-2 (FABP2) Ala54Thr polymorphism results in serum saturated fatty acids elevating. In the present study, we assessed the association of FABP2 gene polymorphism (Ala54Thr) with DR in Chinese population.

**Materials/Methods:**

In this case–control association study, 810 T2DM patients were recruited. 420 patients with retinal neovascularization, microneurysms and hemorrhages were considered as cases (DR) and 390 patients with T2DM and no clinical signs of retinopathy (DNR), were recruited as controls. Genotypes for FABP2(Ala54Thr) polymorphisms were assessed with the PCR-RFLP method.

**Results:**

A significant difference in genotype distribution and allele frequency was observed between cases and controls. Patients with DR had significantly higher frequency of the Ala/Thr + Thr/Thr genotypes compared to DNR group [62.6% vs. 46.2%; OR (95% CI), 1.95 (1.48-2.59); p < 0.001]. The DR group showed a significantly higher frequency of the the Thr allele compared to the DNR group [39.5% vs. 29.4%; OR (95% CI), 1.56 (1.16-2.09); p = 0.003]. Binary logistic analyses showed FFA levels (*p* = 0.014) and Ala54Thr (*p* = 0.011) were independent correlates of the presence of DR.

**Conclusions:**

We examined that FABP2 polymophism on the Ala54Thr is significant and independent associated with DR.

## Introduction

Diabetic retinopathy (DR) is a major microvascular complication affecting the retina and a leading cause of blindness worldwide [1]. Endothelial dysfunction which could cause the damage of the blood-retinal barrier is one of pathogenesis [2]. DR and diabetic nephropathy (DN) are both the microvascular complications of type 2 diabetes (T2D). They have the similar pathogenesis. In clinical, patients who have DN usually have different levels of DR. Canani LH etal’s study showed that the fatty acid-binding protein-2 (FABP2) Ala54Thr polymorphism was associated with renal disease in patients with T2D [3]. The intestinal fatty acid binding protein-2 (FABP2) gene codes a protein expressed in enterocytes and is responsible for the absorption of long-chain fatty acids [4,5]. A single nucleotide polymorphism (SNP) in the FABP2 gene at codon 54 causes anamino acid change (Ala → Thr). This change affects the ability of the protein to bind and transport dietary fatty acids especially saturated fatty acids. Serum saturated fatty acids might induce endothelial dysfunction which are related to increased microvascular mortality [6]. Previous studies demostrated FABP2 polymorphism is associated with T2D [7]. Based on these observations, we hypothesized that genetically predisposed diabetic patients exposed to a diet with a high content of saturated fatty acids might be at high risk for developing DR as developing DN. Therefore, we examined whether FABP2 polymophism on the Ala54Thr which is functionally altered FABP2 protein is associated with DR.

## Materials and methods

### Subjects

In this case control association study, 810 individuals with confirmed T2DM who were recruited from Tianjin Metabolic Diseases Hospital (Tianjin, China). Diabetes was diagnosed according to the World Health Organization (WHO) criteria. The research protocol was approved by the local ethics committee and informed written conset was obtained from each subjects. Ophthalmic examination that included visual acuity testing, Humphrey’s perimetry, ocular coherence tomography and fundus examination followed by fundus photography, was conducted on all individuals. The evaluation of DR was according to the diagnostic criteria of the American Academy of Ophthalmology (AAO) 2001 Annual Meeting. Among the 810 subjects with DM, there were 420 patients with DR (cases) and 390 patients without evidence of retinopathy (DNR) (controls). Information such as age, sex, weight, height, body mass index (BMI), age of onset of diabetes mellitus, fasting glucose levels, duration of diabetes mellitus, any other associated anomalies and blood pressure were collected from on a pre-designed questionnaire.

### Biochemical analysis

Blood samples were obtained after an overnight fast. the concentrations of TG (Vitros TRIG DTD, Johnson & Johnson, New Brunswick, NJ, USA), total cholesterol (TC; Vitros CHOL DTD, Johnson & Johnson), and glucose (Vitros GLU DTD, Johnson & Johnson) were analyzed with a Vitros Chemistry DT60 II (Johnson & Johnson) using slides. FFA (Nefa HR kit, Wako, Osaka, Japan) was analyzed using a Hitachi 7180 (Hitachi, Tokyo, Japan).

### DNA analysis

Genomic DNA was prepared from white blood cells using the Puregene® DNA Isolation kit (Gentra Systems, Inc.). The genotyping of the *Ala54Thr* polymorphism was performed by PCR amplification as previously described [3,7]. For all samples, reading of the genotype was independently carried out by two individuals.

### Statistical analysis

Statistical analysis was performed using the Statistical Package for Social Sciences (SPSS 18.0 for windows, SPSS Inc., Cnicago, IL, USA). Allelic frequencies were calculated by a gene-counting method. Agreement with Hardy-Weinberg expectations was tested using a χ^2^ goodness-of-fit test. Comparison of the means between the 2 groups was analyzed by Student *t* test. The χ^2^ test was used to compare the proportions of genotypes or alleles. Odds ratio (OR) and 95% confidence intervals (CI) were calculated models for systolic blood pressure, diastolic blood pressure, FFA levels and genotypes were fitted. A two-sided probablity value of less than 0.05 was considered to indicate statistical significance.

## Results

The clinical and biochemical characteristics of the study population are shown in Table 1. There were no significant differences in the age, TC levels, diastolic blood pressure and gender distribution between DR (cases) and DNR (controls) groups. The DR group had a higher prevalence of duration of diabetes (*p* < 0.001), systolic blood pressure (*p* < 0.001), fasting glucose (*p* < 0.001), HbA1c levels (*p* < 0.001), TG levels (*p* < 0.001), BMI (*p* = 0.002) and FFA levels (*p* < 0.001) compared with the DNR group.

**Table 1:** **Clinical and biochemical characteristics of the study population**

**characteristics**	**Diabetic retinopathy (n = 420)**	**Diabetic without retinopathy (n = 390)**	**P value**
Sex(Male/Female)	212/208	190/200	>0.05
Age(yrs)	55.4 ± 8.9	56.3 ± 8.6	0.072
Duration of diabetes (years)	15.5 ± 2.3	13.3 ± 2.8	<0.001
Systolic blood pressure (mm Hg)	150 ± 17	140 ± 13	<0.001
Diastolic blood pressure (mm Hg)	83 ± 13	82 ± 9	0.104
Fasting glucose (mmol/l)	7.6 ± 2.6	6.7 ± 2.1	<0.001
HbA1c(%)	9.3 ± 2.5	8.2 ± 2.4	<0.001
Total cholesterol (mmol/l)	5.2 ± 1.1	5.1 ± 0.9	0.078
Triglycerides (mmol/l)	2.4 ± 1.1	1.8 ± 0.4	<0.001
Free fatty acid (mEq/l)	1.8 ± 0.3	1.3 ± 0.3	<0.001
BMI(kg/m2)	27.1 ± 3.3	26.4 ± 3.5	0.002

The genotype distribution and the relative allele frequency of the Ala54Thr polymorphism at the FABP2 gene in DR and DNR groups are shown in Table 2. Genotype frequencies did not deviate from the Hardy-Weinberg equilibrium in DNR group (χ^2^ = 2.97; *p* = 0.226) and DR group (χ^2^ = 4.89 ; *p* = 0.086). A significant difference in genotype distribution and allele frequency was observed between DR and DNR groups. DR group had significantly higher frequency of Ala/Thr + Thr/Thr genotypes compared to DNR group [62.6% vs. 46.2%; OR (95% CI), 1.95 (1.48-2.59); p < 0.001]. The DR group showed a significant higher frequency of the Thr allele compared to the DNR group [39.5% vs. 29.4%; OR (95% CI), 1.56 (1.16-2.09); p = 0.003].

**Table 2:** **Genotypes and allele frequencies of the FABP2 pholymorphism in diabetic retinopathy (cases) and diabetic without retinopathy (control) groups**

	**Diabetic retinopathy (n = 420)**	**Diabetic without retinopathy (n = 390)**	**P value**	**OR (95%CI)**
**Genotype**				
**Ala/Ala**	37.4% (157)	53.8% (210)		
**Ala/Thr**	46.1% (194)	39.7% (155)	<0.001	1.98 (1.38-2.89)
**Thr/Thr**	16.5% (69)	6.5% (25)		
**Allele frequency (%)**				
**Ala**	60.5%	70.6%		
**Thr**	39.5%	29.4%	0.003	1.56 (1.16-2.09)

We used binary logistic regression to test for independent correlates of the presence of DR. Included in the model were systolic blood pressure, diastolic blood pressure, HbA1C levels, FFA levels and Ala54Thr FABP2 polymorphism. Systolic blood pressure (*p* < 0.001), HbA1c levels (*p* < 0.001), FFA levels (*p* = 0.014) and Ala54Thr (*p* = 0.011) were independent correlates of the presence of DR (Table 3).

**Table 3:** **Binary logistic regression modeling using diabetic retinopathy as a dependent variable**

**Variables**	**Beta-coefficient**	**SE**	**P value**	**OR (95% CI)**
54Ala/Thr polymorphism	0.561	0.226	0.012	1.75 (1.12-2.73)
Systolic blood pressure	0.986	0.241	<0.001	2.68 (1.67-4.30)
Duration of diabetes	0.956	0.236	<0.001	1.44 (0.91-1.94)
BMI	0.218	0.218	0.317	1.24 (0.81-1.91)
HbA1c (%)	1.614	0.226	<0.001	5.01 (3.22-7.83)

## Discussion

Type 2 diabetes as well as its complication is a multifactorial disease in which genetic and environmental factors play a great role [8]. These factors may differ in each race or ethnic group. In these patients with type 2 diabetes, the presence of the Ala54Thr polymorphism of the FABP2 gene was assciated with diabetic retinopathy in Chinese. To our knowledge, this is the first time this association was been reported.

Diabetes damages small blood vessels throughout the body, leading to reduced blood flow. When these changes affect the tiny blood vessels in the eye, diabetic retinopathy may occur [9]. Endothelial dysfuction is one of damages of small and tiny blood vesesels [2]. Saturated fatty acids might be related to endothelial dysfunction [10]. In our study, we showed that the FFA levels of DR group were significantly higher than DNR group which has the similar result with a study that patients with type 2 diabetes and microalbuminuria had higher levels of saturated fatty acids, especially stearic and palmitic acids, than normoalbuminuric patients [11]. Microalbuminuria is the ealrly symptom of diabetic nephropathy which belongs to diabetic microangiopathy as well as DR. Therefore they have the similar pathogenesis which is endothelial dysfuction.

The Ala54Thr polymorphism of FABP2 gene has been associated with an increase in FABP2 affinity for long-chain fatty acids such as stearic and palmitic acids which are saturated fatty acids and consequently with higher levels of these fatty acids after a test meal [12]. We found an excess of Ala/Thr + Thr/Thr genotypes for the Ala54Thr variant among DR group compared to DNR group and in addition, Ala54Thr FABP2 polymorphism was the independent correlate of the presence of DR. A previous study has reported The Ala54Thr polymorphism of FABP2 gene was associated with DN [3]. The Ala54Thr polymorphism of FABP2 gene may lead to saturated fatty acids increasing which can cause endothelial dysfuction and then triggers DR and DN developing.

In summary, FABP2 polymophism on the Ala54Thr which is functionally altered FABP2 protein function is significant and independent associated with DR.

## Abbreviations

SNP: Single nucleotide polymorphism
PCR-RFLP: Polymerase chain reaction-based restriction fragment length polymorphism
